# What do the fundamental constants of physics tell us about life?

**Published:** 2025-09-11

**Authors:** Pankaj Mehta, Jané Kondev

**Affiliations:** 1 Department of Physics and Faculty for Computing and Data Science, Boston University, Boston MA & 02215, USA.; 2 Martin A. Fisher School of Physics, Brandeis University, Waltham MA & 02453, USA.

## Abstract

In the 1970s, the renowned physicist Victor Weisskopf famously developed a research program to qualitatively explain properties of matter in terms of the fundamental constants of physics. But there was one type of matter prominently missing from Weisskopf’s analysis: life. Here, we develop Weisskopf-style arguments demonstrating how the fundamental constants of physics can be used to understand the properties of living systems. By combining biophysical arguments and dimensional analysis, we show that vital properties of chemical self-replicators, such as growth yield, minimum doubling time, and minimum power consumption in dormancy, can be quantitatively estimated using fundamental physical constants. The calculations highlight how the laws of physics constrain chemistry-based life on Earth, and if it exists, elsewhere in our universe.

A defining achievement of twentieth century physics is the realization that the properties of matter can be understood using the fundamental theories of physics. Features as diverse as the density of rocks, the visibility of compact matter, and the size of mountains, all emerge from quantum mechanical interaction between electrons, nuclei, and electromagnetic radiation. Motivated by this observation, more than fifty years ago, the physicist Viki Weisskopf developed an influential research program showing that it was possible to semi-quantitatively understand the properties of terrestrial matter in terms of six fundamental constants: the speed of light c, Planck’s constant ℏ (the constant governing quantum fluctuations), the charge of an electron e, the mass of the electron $m_{e}$, the mass of the proton $m_{p}$, and Netwon’s gravitational constants $G_{N}$. For example, using clever physical argumentation Weiskopf famously provided order-of-magnitude estimates for the maximum height of mountains on Earth (~ 26 km) and Mars (~ 50 km) (1,2). Weisskopf argued that such qualitative arguments complement more rigorous quantitative calculations by highlighting the essential physics needed to explain a phenomenon.

Weisskopf applied his program of explaining the world in terms of fundamental physical constants to all kinds of matter ranging from rocks, to water waves, to stars. However, there was one prominent form of matter missing from Weisskopf’s original analysis: living systems. From a physical perspective, life is a novel form of non-equilibrium self-organized matter whose defining feature is high-fidelity self-replication (3). This process requires living organisms to carry out two general classes of metabolic tasks: extracting energy from the environment (catabolism) and synthesizing the new material needed for self-replication (anabolism) (4–6) (see Figure 1).

In chemistry-based life like that found on Earth, self-replication is powered by transferring electrons from a high-energy state to a lower-energy final state. For example, in aerobic respiration electrons in high-energy molecules like sugars are transferred to low-energy oxygen molecules. This observation led Noble-prize winning biochemist Albert Szent-Gyorgi to quip that ”Life is nothing but a high energy electron looking for a place to rest” (7, 8). This reduction of life to electrons of course misses much of what makes living systems so interesting – the central role played by information transmission, ecology, evolution, and complexity (9–11). Nonetheless, it has the virtue of highlighting the fact that the law of physics, and in particular quantum mechanics and thermodynamics, still fundamentally constrain life. This suggests it should be possible to extend Weisskopf-style argumentation to understand living matter (12).

In order to carry out such a program, we draw on the rich emerging literature quantifying biological phenomena across living systems (13–15). For concreteness, we focus here on three properties of chemical self-replicators: (1) the growth yield Y which measures the amount of biomass produced (in grams) per amount of energy consumed; (2) the minimum time for self-replication; and (3) the minimum power consumption $P_{min}$ needed by a dormant cell in order not to die (see Figure 1).

For example, as discussed by (6), for a wide variety of single-cell organisms the growth yield in energy-limited conditions is about $Y\sim 1\times 10^{-4}$ g J^−1^. This number is often reported as Y~5–10 g (mol ATP.)^−1^. To convert between moles ATP and Joules, we have used the fact that 1 mol ATP yields ~ 50kJ in standard conditions. Similarly, a mix of theoretical arguments and empirical measurements suggest that the fastest self-replication time for a bacterial cell is approximately $\tau_{double}\approx 5\times 10^{2}$ s (12, 14). In contrast, bacteria in extreme environments have doubling times that are almost eight orders of magnitude larger (16–18). Can we understand the origin of these numbers in terms of fundamental physical constants? Empirical measurements also suggest that the minimum maintenance power of a culture of dormant cells is approximately 10^−2^ mol ATP g^−1^ hr^−1^ which, using the fact that a typical bacterial cell weighs 10^−12^ g, translates to a maintenance power per cell of $P_{min}\approx 10^{-13}$ J/s/cell (14, 19). More recent measurements suggest a slightly higher maintenance power $P_{min}\approx 10^{-11}$ J/s/cell (20). How do the laws of physics give rise to this?

## Physical scales and fundamental constants

Our starting point for understanding these numbers is the fact that the physical processes underlying both catabolism and anabolism in chemical self-replicators are governed by quantum mechanical interactions between electrons and nuclei. These interactions are associated with a characteristic energy scale, the Rydberg energy $Ry\approx 13.6\;\text{eV}\approx 2.18\times 10^{-18}$ J, and a characteristic length scale, the Bohr radius $a_{0}\approx 0.53\text{Å}\approx 5.3\times 10^{-11}$ m. Weisskopf gave a simple qualitative argument for estimating these scales using energy minimization and the de Broglie relation, p=ℏ/λ, which relates the momentum of a particle, p, to its wavelength, λ, through Planck’s constant $\hbar =1.05\times 10^{-34}$ J·s (2).

The energy of an electron with momentum p confined within a atom of radius r can be written as a sum of the electron’s kinetic energy and potential energy due to electromagnetic interactions $E=\frac{p^{2}}{2m_{e}}-\frac{1}{4\pi \epsilon_{0}}\frac{e^{2}}{r}$, where $\epsilon_{0}$ is the vacuum electric permittivity, e is the electron charge, and $m_{e}$ is the mass of the electron. Since a particle confined to a region of size r will have a wavelength λ~r, we can use the de Broglie relation to write the characteristic momentum associated with a confined electron, namely p~ℏ/r. Substituting this into the expression above yields the energy of the electron as a function of the confinement distance,

(1) $$E(r)\approx \frac{\hbar^{2}}{2m_{e}r^{2}}-\frac{1}{4\pi \epsilon_{0}}\frac{e^{2}}{r}.$$

The electron is typically located at a distance that minimizes its total energy, allowing us to calculate the Bohr radius $a_{0}$ by finding the value of r that minimizes E(r). A straight forward calculations yields (see Methods)

(2) $$a_{0}=\frac{4\pi \epsilon_{0}\hbar^{2}}{m_{e}e^{2}}=\frac{\hbar }{\alpha m_{e}c},$$

where in going to the second expression we have introduced the fine structure constant α≈1/137, the speed of light, c, and used definition of the vacuum electric permittivity $\epsilon_{0}=\frac{e^{2}}{4\pi \alpha \hbar c}$. Substituting $a_{0}$ into Eq. 1 gives us the typical energy associated with an electron at a distance $a_{0}$, the Rydberg energy

(3) $$Ry=-E\left(a_{0}\right)=\frac{e^{2}}{8\pi \epsilon_{0}a_{0}}=\frac{1}{2}\alpha^{2}m_{e}c^{2}.$$

The Rydberg energy, Ry, and the Bohr radius, $a_{0}$, are the fundamental length and energy scales associated with chemical physics.

We can also use the Rydberg energy to estimate the energy, $E_{bond}$, associated with breaking a chemical bond. To do so, we exploit the empirical observation that the size of a chemical bond $l_{bond}$ is generally a few Bohr radii so that we can write $l_{bond}\sim f_{bond}\;a_{0}$. We adopt the notation that singles out the dimension-full quantity $\left(a_{0}\right)$ that sets the order of magnitude of the quantity of interest and clump the rest into a numerical factor, $f_{bond}$. For example, for a carbon-carbon bond, $f_{bond}\approx 3$. Since the chemical bond corresponds to ”shared” electrons between atoms, we can estimate its energy by the Coulomb energy of an electron whose distance to the positively charged nucleus is approximately equal to the bond length (see Eq. 3). From this simple physical picture we can immediately conclude that the bond energy scales inversely with the bond length, i.e., $E_{bond}\sim Ry/f_{bond}$. For $f_{bond}=3$, this expression yields $E_{bond}\approx 4.5$ eV which is comparable to the empirically measured energy to break a C-C bond, 350 kJ/mol or 3.6 eV/bond.

The physics of life is not governed solely by quantum mechanics. Instead, it emerges from a delicate interplay between thermal and quantum fluctuations (21). The reason for this is that self-replication is an inherently non-equilibrium phenomena that has a definitive arrow of time. The physical constant governing the scale of thermal fluctuations is the Boltzmann constant, $k_{B}=1.4\times 10^{-23}$ J·K^−1^. We can characterize the importance of thermal fluctuations at a temperature T in terms of the thermal energy, $k_{B}T$. At standard temperatures relevant to life on earth (T=298K), $k_{B}T=4\times 10^{-21}$ J or in more relevant units for us, 25.7 meV. Notice that the thermal energy scale $k_{B}T$ is more than 500 times smaller than the Rydberg energy, Ry, and about 100 times smaller than $E_{bond}$. This vast difference in energy scales between quantum mechanics and thermal energy underlies the extreme stability of chemical bonds and explains why chemical reactions must be actively catalyzed in living systems.

Thus, far we have focused on identifying relevant energy and length scales. However, self-replication is a dynamics process that also requires us to understand the time scales of chemical processes. The reaction rates of chemical reactions are limited by the ability of molecules to find each other through diffusion in their fluid environment. In the diffusion limited regime, a molecule of size r at a concentration c encounters a target of size R at a rate $k_{on}=4\pi DcR$, where D is the diffusion constant (22). We show below that there is a maximum rate $k_{on}^{max}$ at which molecules can find a target. For a molecule diffusing in a fluid of concentration $c_{f}$ with atomic number $A_{f}$ and temperature T, we have

(4) $$k_{on}^{max}=A_{f}^{-\frac{1}{2}}\left(\frac{4\pi }{3}\right)\times \left(\frac{c}{c_{f}}\right)\times \left(\frac{R}{r}\right)\times \tau_{min}^{-1}(T)$$

where we have defined the characteristic “kinetic time”

(5) $$\tau_{min}(T)=\frac{\hbar }{k_{B}T}{\left(\frac{m_{e}}{m_{p}}\right)}^{-\frac{1}{2}}.$$

At 37° C, $\tau_{min}$ is about 1 picosecond. For molecules at a concentration of 100mM in water, like amino acids in the cytoplasm, the numerical value of $k^{max}=2\times 10^{9}$ s^−1^. In calculating this, we have used the fact that for water $A_{f}=18$ and $c_{f}=55\text{M}$. Amusingly, the dominant numerical contribution to $\tau_{min}$ comes from the ratio $\hbar /k_{B}T$, highlighting how chemical kinetics emerges from a subtle interplay between thermal and quantum fluctuations. As expected, for higher temperatures thermal fluctuations get stronger and this time scale gets smaller.

A full derivation of the expression in Eq. 4 is given in the Methods. Here, we briefly outline the physical intuition underlying this expression. Our starting point is the observation that we can re-express the diffusion constant in terms of the temperature T and the fluid viscosity η using the Stokes-Einstein relation $D=\frac{k_{B}T}{6\pi \eta r}$. Rather than work with the viscosity directly, it is useful to rewrite this relation in terms of the kinematic viscosity, $v=\eta /\rho_{f}$, where $\rho_{f}=A_{f}m_{p}c_{f}$ is the mass density of the fluid. In general, both η and v are a function of temperature. However, Ed Purcell pointed out that varied fluids all exhibit a similar numerical value for the minimum kinematic viscosity $v_{min}$ (23,24). Surprisingly, $v_{min}$ does not depend on temperature and is entirely a property of quantum mechanical interactions between fluid molecules. The natural scale for $v_{min}$ is set by a combination of fundamental constants we call the Berg viscosity, $v_{B}=\frac{1}{2\pi }\frac{\hbar }{\sqrt{m_{e}m_{p}}}$, with $v_{min}=A_{f}^{-1/2}v_{B}$ ((24) and Methods). Since $k_{on}$ is inversely proportional to v, substituting $v_{min}$ into the expression for $k_{on}$ allows us to calculate the fastest possible kinetic rate, $k_{on}^{max}$, resulting in Eq. 4. Finally, we note that we can express $\tau_{min}(T)$ in terms of the the Berg viscosity as $\tau_{min}(T)/(2\pi )=m_{p}v_{B}/k_{B}T$.

## Properties of chemical self-replicators

**Figure 2:**
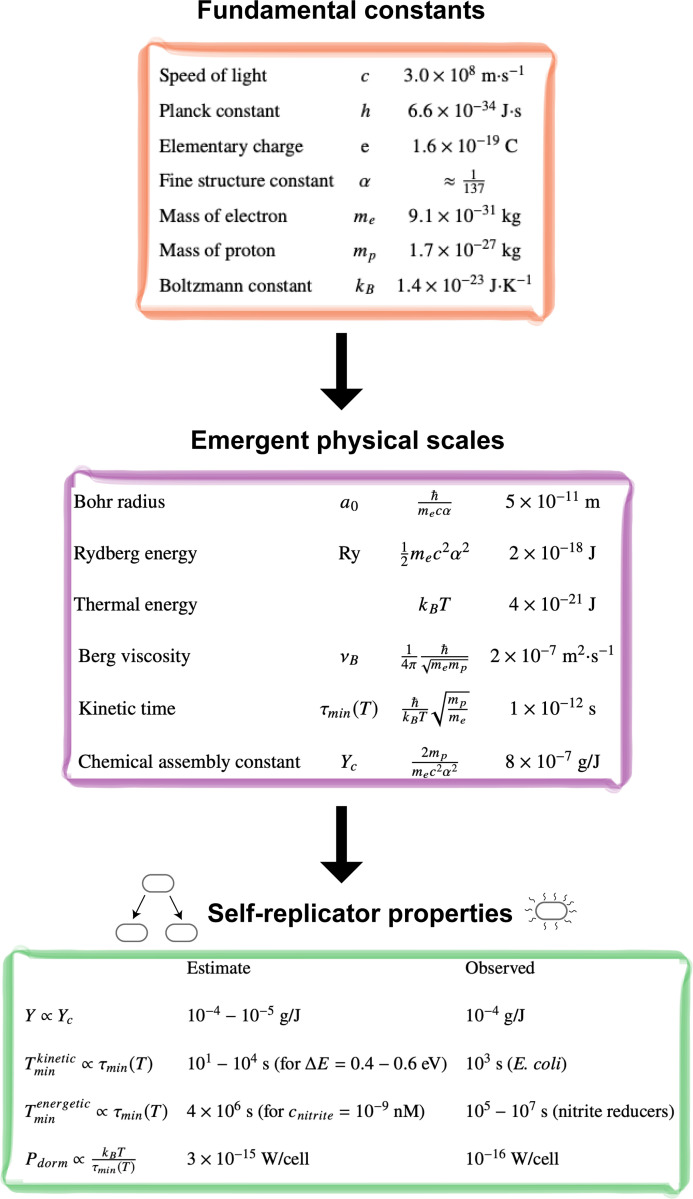
From fundamental constants to self-replicator properties. The fundamental physical constants give rise to emergent physical scales that govern the properties of chemical self-replicators.

The relevant fundamental physical constants and emergent physical scales are summarized in Figure 2. We now show how these quantities can be used to quantitatively estimate the properties of living systems

### Mass yield

A defining property of life is self-replication. This requires organisms to extract energy from their environments in order to create new biomass. One interesting quantity that we can use to characterize this process is the mass yield, Y, which measures the amount of new mass produced by the organism per unit of energy consumed. As discussed above, for a wide variety of single-cell organisms, experimental measurements suggest that $Y\sim 1\times 10^{-4}$ g J^−1^. How can we understand this quantity in terms of fundamental physical scales discussed above?

Our starting point is observation that the dominant source of energy consumption during self-reproduction are anabolic processes that synthesize new biomass. Consider an organism of dry mass M that requires the synthesis of $N_{bond}$ bonds at an energetic cost $E_{bond}=Ry/f_{bond}$. If the synthesis of these new bonds is the dominant source of energy consumption, the mass yield can be written as $Y=M/\left(N_{bond}E_{bond}\right)=f_{bond}M/\left(N_{bond}Ry\right)$. Generically, the number of new bonds that need to be formed will be proportional to the number of atoms N in the organism, so that $N_{bond}\approx bN$, with b a numeric proportionality constant. The number of atoms N and the mass M are related through the average molecular weight of the biomolecules, namely, $M=NA_{bio}m_{p}$ where $m_{p}$ is the mass of a proton and $A_{bio}$ is the average atomic mass of constituent biomass molecules. Rearranging and re-expressing the Rydberg energy Ry in terms of fundamental constants, yield the expression

(6) $$Y\approx \left(\frac{A_{bio}f_{bond}}{b}\right)\times Y_{c},$$

with

(7) $$Y_{c}=\frac{2m_{p}}{m_{e}c^{2}\alpha^{2}}.$$

$Y_{c}$ defines a new physical scale we call the chemical assembly constant with a numerical value ~ 8 × 10^−7^ g/J. A striking property of this expression is that it depends only on the composition of an organism and not on its size or mass.

We can compare the predictions of this expression to experimental observations in archaea and bacteria. To do so, we makes use of the fact that life on earth is composed largely of carbon so that $A_{b}\approx 13$ and $f_{bond}\approx 3$. We can also estimate b by exploiting the fact that the bulk of newly synthesized biomass are proteins. Proteins are composed of amino acids, which range in size from 10–30 atoms. Depending on the medium in which cells are grown, amino acid synthesis can require anywhere from 0 bonds (i.e. all amino acids are provided in the medium) to 15–25 bonds in rich medium. In addition, making proteins requires synthesizing one peptide bond per amino acid. This means that b~1 in minimal media and b~1/10 in rich media. For these choices, we have that $Y\approx 3\times 10^{-5}$ g/J and $Y\approx 3\times 10^{-4}$ g/J, respectively These estimates are compare favorably to the experimentally observed values of 1 × 10^−4^*g*/*J*.

### Time for replication

Wenowchangeourfocusfromenergeticstodynamics.Single-celledorganismsexhibitanincredibly wide range of doubling times. *E. coli* in rich media doubles in 20 minutes, whereas ammonia-oxidizing archaea take a day to several months to self-replicate, with the exact time depending on environmental conditions (16). Lithoautotrophic bacteria in the deep sea crust have even longer doubling times, estimated to be of order years (18). To understand the physical origins of these numbers, it is helpful to distinguish between two possible scenarios. The first is that growth is kinetically limited. In this case, the cell division time is limited by the time it takes to carry out the chemical reactions needed to synthesize new biomass (12). The second possibility is that self-replication is energy-limited. In this case, the division time is set by the time it takes to extract sufficient energy from the environment to create a new organism (6).

For kinetically limited replicators, it is essential to estimate the time scale associated with chemical reactions. To do so, we will make use of transition-state theory in chemical kinetics. Within this framework, the rate of a chemical reaction is simply the product of the diffusion limited on rate, $k_{on}^{max}$, and an Arrhenius factor, $e^{\frac{-\text{Δ}E}{k_{B}T}}$, stemming from the fact that the reactions cannot happen spontaneously and must pass through a high-energy intermediate transition state. If the transition state has energy ΔE, then

(8) $$k^{max}=k_{on}^{max}e^{\frac{-\text{Δ}E}{k_{B}T}},$$

with $k_{on}^{max}$ given by Eq. 4.

To calculate $T_{min}$, we need an estimate of the activation energy ΔE of the transition state. One qualitative way of estimating ΔE is to model chemical bonds as springs and ask how much bonds are stretched or compressed at the transition state (see Methods). The “spring constant” of a bond of length $l_{bond}=f_{bond}a_{0}$ can be estimated from the bond energy using Hooke’s law, $E_{bond}=K_{bond}l_{bond}^{2}/2$. Using the fact that $E_{bond}=Ry/f_{bond}$, we can write the spring constant as function of the Rydberg energy as $K_{bond}=2Ry/\left(f_{bond}l_{bond}^{2}\right)$. At the transition state, the bond length changes by Δl. Let us denote the fractional change in the length of the bond by $\delta =\text{Δ}l/l_{bond}$. The activation barrier of the transition state is $\text{Δ}E^{min}=\frac{1}{2}K_{bond}{\left(\text{Δ}l\right)}^{2}=\delta^{2}Ry/f_{bond}$. For $f_{bond}=3$ and δ=0.3−0.5 – a reasonable choice for a wide variety of reactions – one gets that ΔE≈0.4−1.1 eV. This is comparable to the observed activation energy for most common reactions.

We are now in a position to calculate the minimum doubling time $T_{min}$ for kinetically limited self-replicator. The fastest self-replicator is an organism where each molecule replicates itself (12). If a molecule on average has $N_{M}$ bonds that need to be synthesized, then the minimum doubling will simply be

(9) $$T_{min}=N_{M}/k^{max}=N_{M}\times A_{f}^{\frac{1}{2}}\left(\frac{4\pi }{3}\right)\times \left(\frac{c_{f}}{c}\right)\times e^{\frac{\delta^{2}}{f_{bond}}\frac{Ry}{k_{B}T}}\times \tau_{min}(T).$$

In writing this, we have used the fact that the molecules are of roughly same size so that R≈r in Eq. 4.

For *E. coli* growing in exponential growth at 37° C, the biomass production of the cell is dominated by the production of new ribosomes which account for the bulk of the dry mass (5). A good approximation to this situation is to assume that every protein translates itself (12). In this case, we can choose N to be equal to the typical number of peptide bonds in all ribosomal proteins, $N_{M}\sim 7400$. The typical concentration c of amino acid precursors is 100 mM resulting in an $k_{on}^{max}=2\times 10^{9}$ s^−1^. For ΔE in the range 0.4 − 1.1eV, this give $k^{max}$ in the range 10^2^ s^−1^ to 10^−10^ s^−1^, reflecting the extreme sensitivity of this rate to the activation barrier. Using these rates, one finds that $T_{min}\approx 10s$ for ΔE=0.4 eV, $T_{min}\approx 480s$ for ΔE=0.5, and $T_{min}\approx 10^{11}\text{s}$ for ΔE=1 eV.

These numbers can be compared to experiments. The fastest known translation rate is about 20 amino acids per second, corresponding to a $k_{on}^{max}=20$ s^−1^. The fastest observed doubling time is for the bacterium *Vibrio natriegens*, which has a $T_{min}\approx 600$ s. While our estimates span a wide range, they are consistent with these experimental observations and put a strong lower bound on the fastest doubling times. However, the exponential dependence of chemical kinetic constants on activation energy make it difficult to make more stringent predictions for kinetically-limited self-replicators using purely qualitative methods.

In energy-poor environments, self-replication is not limited by kinetics, but by the ability of organism to harvest electron donor molecules from the environment. If we denote the rate at which bacteria can extract energy from the environment by $P_{E}$, then we can qualitatively estimate the division time for energy limited replicators using the formula $T_{min}=E_{tot}/P_{E}$, where $E_{tot}=N_{bond}E_{bond}$ is the total energy needed for an organism to copy itself. In writing this last expression, we have once again assumed that the dominant energy expenditure is the synthesis of new bonds. It is helpful to once again express $N_{bond}$ in terms of the mass of an organism, namely $N_{bond}=\frac{bM}{A_{bio}m_{p}}$, with b the average number of bonds per atom.

In order to estimate $P_{E}$, we model bacteria as absorbing spheres of size R≈1 micron that capture elector donor molecules found in the environment at a concentration c of size r≈1 nm. The rate at which bacteria can extract energy is limited by the diffusion limited flux incident on the cell, which can be calculated from Eq. 4. For nitrite reducing bacteria, c is typically in the nM range whereas for deep sea chemolithoautotrophs, the limiting source of energy is dissolved $H_{2}$ molecules which are found at sub-nanomolar concentrations, $c\approx 10^{-10}-10^{-12}$ M. If organisms can extract an energy $E_{redox}$ per molecule, the power harvested from the environment is $P^{max}=k_{on}^{max}E_{redox}$. $E_{redox}$ is set by the details of redox chemistry and is typically of order 0.1 − 1*eV*. Combining these expressions and using the fact $E_{bond}=Ry/f_{bond}$, one has

(10) $$T_{min}=A_{f}^{\frac{1}{2}}\left(\frac{3\pi bA_{bio}}{4f_{bond}}\right)\times \left(\frac{c_{f}}{c}\right)\times \left(\frac{r}{R}\right)\times \left(\frac{M}{m_{p}}\right)\times \left(\frac{Ry}{E_{redox}}\right)\times \tau_{min}(T)$$

Using a value of $M=10^{-12}$ g and T≈300K, we arrive at an estimated doubling time of $T_{min}=4\times 10^{6}\text{s}$ or 42 days for nitrite reducers in and $T_{min}=4\times 10^{7}-4\times 10^{9}\text{s}$ or 1–100 years for bacteria that live deep in the ocean crust, consistent with experimental observations (16–18).

### Power consumption of dormant cells

Thus, far we have focused on the energetics and kinetics of reproduction. However, cells must expend energy even when they are not growing (25). The reason for this is that life is inherently a non-equilibrium phenomena that requires energy in order to counteract entropic forces. Paraphrasing Schrondinger, life feeds on “negative entropy” (3). For compartmentalized organisms with a membrane, this process is dominated by the need to maintain the cellular membrane potential $V_{m}$ resulting from concentration differences of charged ions inside and outside the cell (25,26). Even though the membrane is an excellent insulator it still supports a ”leakage” ion current across the membrane. This leads to loss of membrane potential which cells actively counteract by using energy-consuming ion pumps.

The simple idea we fully explore in the Methods, is that thermal fluctuations open pores in the membrane which allow ions to pass through. There we show that this requires the cell to consume energy at a rate

(11) $$P_{dorm}=\frac{1}{3}A_{f}^{-\frac{1}{2}}\times \left(\frac{c_{o}}{c_{f}}\right)\times n_{pore}\times \left(\frac{S_{p}}{d_{m}r}\right)\times {\left[\ln\left(\frac{c_{o}}{c_{i}}\right)\right]}^{2}\times \frac{k_{B}T}{\tau_{min}(T)},$$

where $n_{pore}$ is the expected number of pores formed due to thermal fluctuations, $S_{p}$ is the surface area of the membrane pore, which we take to have the radius that is typical of a hydrated ion (i.e., a few Å); $d_{m}$ is the membrane thickness, r is the size of an ion, and $c_{o/i}$ is the concentration of charged ions outside/inside the cell. Notice that the fundamental physical constants are all contained in the term, $k_{B}T/\tau_{min}(T)$, which has a numerical value of 4 × 10^−9^ J/s at room temperature. This suggests that natural physical scale characterizing entropy production in living system is simply thermal energy $k_{B}T$ divided by the kinetic time scale $\tau_{min}(T)$.

We briefly outline the physical origins of this expression. A full derivation is provided in the Methods. As discussed above, the membrane voltage $V_{m}$ gives rise to a leak current I across the membrane. From elementary physics, we know that the power dissipated by this current is just $P=IV_{m}$. Due to the second law of thermodynamics, cell must expend at least this much energy per unit time to counteract leak currents. For this reason, the problem of calculating $P_{max}$ reduces to the deriving expressions for $V_{m}$ and the maximum possible leak current $I^{dorm}$.

The membrane potential can be calculated using standard thermodynamic arguments. The electrical potential energy due to $V_{m}$ must equal to the free energy resulting from concentration differences, resulting in the identity $V_{m}=\frac{k_{B}T}{ze}\ln\left(\frac{c_{o}}{c_{i}}\right)$, where z is the ion charge. The prefactor $k_{B}T/e$ is often called the thermal voltage and is approximately 26 mV at room temperature (T=300K) (15). For a membrane of thickness $d_{m}$, this voltage difference gives rise to an electric field of strength $E_{m}=V_{m}/d_{m}$ across the membrane. The resulting force accelerates the ions to a velocity $v_{i}$ at which point the force due to $E_{m}$ is balanced by the frictional drag force on ions from the surrounding fluid, $zeE_{m}=\gamma v_{i}$, with γ the drag coefficient. The corresponding leak current is just the net charge that crosses the membrane per unit time, $I=ze\left(c_{o}-c_{i}\right)S_{p}v_{i}$.

To calculate $v_{i}$, we use the fact that, at steady-state, the electrical and drag forces must balance, resulting in the relation $zeV_{m}/d_{m}=\gamma v_{i}$ (27). For ions of size r, we can relate γ to the kinematic viscosity v using the Stokes relation, $\gamma =6\pi A_{f}C_{f}vr$. Notice that the velocity, and hence the current, will be maximal, when γ, and hence the kinematic viscosity v is at its minimum value. As discussed above, this happens when $v=v_{min}$. This observation allows us to calculate the maximum leak, resulting in Eq. 11.

We can use Eq. 11 to estimate the power consumption of a single membrane pore. Then, by estimating the average number of such pores, $n_{pore}$, that are generated in the cell membrane by thermal fluctuations, we arrive at the power consumption of a dormant cell, $P_{dorm}$. We note that $c_{i}\approx 300$ mM, $d_{m}\approx 4$ nm, r≈0.3 nm, $S_{p}\approx 0.3$ nm^2^, and $\ln\left(\frac{c_{o}}{c_{i}}\right)\approx 3$. Plugging in these numbers gives estimates for the maximal current, $I^{max}\approx 20\times 10^{-12}$, and power dissipation $P_{max}\approx 4\times 10^{-12}$ W for a single membrane pore. To estimate $P_{dorm}$, we simply multiply the power dissipated by a single pore by the expected number of pores $\left(n_{pore}\approx 8\times 10^{-4}\right)$. To make the estimate for $n_{pore}$ we make use of an estimate for the probability of making a pore (≈ 4 × 10^−11^), which we arrive at by estimating the energy cost of a pore (≈ 0.6eV), and by estimating the number of lipids in the inner membrane of an *E.coli* cell (≈ 2 × 10^7^). This gives an estimate $P_{dorm}\approx 3\times 10^{-15}$ W/cell, or 10^3^ ATP/sec/cell, where in going between these numbers we have used the fact that the free energy of ATP hydrolysis in cells is $\text{Δ}G\approx 10^{-19}$ J. This is comparable to recent measurements that estimated the power consumption rate of a cell to be 3 × 10^4^ ATP/s, or equivalently (20). This is also similar to the observed values for dormant bacteria in natural environments (28).

This is remarkably good agreement given the fact that the probability of making a membrane pore is exponentially dependent on the energy cost of pore formation. This means that a small change in the estimate of the pore formation energy such as (ln10) $k_{B}T=0.0057$ eV leads an order of magnitude change in probability of pore formation and equally large change in maintenance cost. Interestingly, data on maintenance energy for bacteria isolated from agricultural and forest soils, marine sediments, as well as polar ice cores, show a much larger variability than the energy requirements for growth, and span at least two orders of magnitude (28). This we would expect based on pore formation energy depending slightly, even at the 10% level, on the lipid composition of the cell membrane.

## Conclusion

Life, like all forms of matter, is subject to the laws of physics. Here, we have provided qualitative arguments showing how many properties of living systems have their origins in fundamental physical laws. Our results suggest that despite the incredible complexity and creativity of evolution, many energetic and kinetic properties of self-replicating organisms can be understood using simple physical arguments from quantum mechanics and thermodynamics. Since the laws of physics are expected to be the same on all planets, we expect that the estimates we provide here should be equally applicable to life on other planets, if its exists. Our expressions also make a strong prediction about the variability of the three quantities we consider (growth yield, minimum doubling time and power consumption in dormancy). Namely, we expect the growth yield to be the most constrained and least variable property of chemical self-replicators across terrestrial and extraterrestrial life. The underlying reason for this is that in contrast to our expressions for the minimum doubling time and power consumption, the growth yield does not have an Arrhenius dependence and hence is not exponentially sensitivity to small changes in activation energies. In the future, it will be interesting to see if such qualitative arguments can be developed further to constrain the physical properties of chemistry-based self-replicators and aid in developing strategies for the search for extraterrestrial life.

## Supplementary Material

Supplement 1

## Figures and Tables

**Figure 1:**
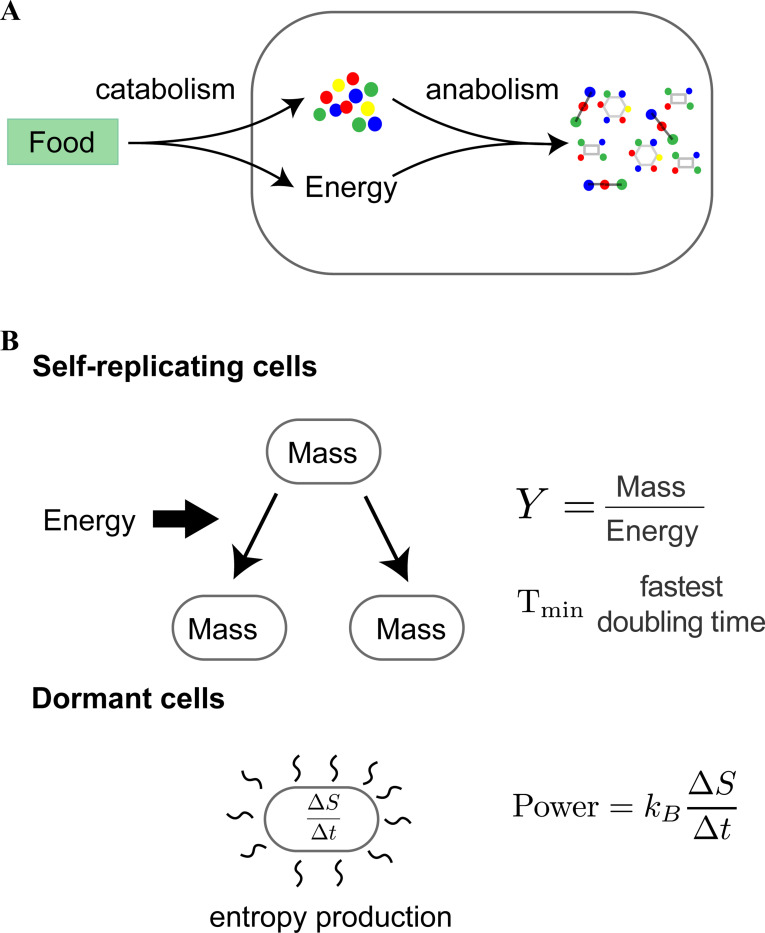
Characterizing chemical self-replicators. (a) Self-replication requires organisms to break down food molecules from the environment to extract energy and metabolic precursors (catabolism) and then use this energy to synthesize the complex molecules necessary for self-replication (anabolism). (b) We focus on three properties of self-replication: (i) the mass per unit energy consumed needed to self-replicate, (ii) the time it takes for self-replication, and (iii) the energy per unit time a dormant cell must expend in order to stay alive and counter entropic forces.
