# Supplementary material for: What do the fundamental constants of physics tell us about life?
Source: ArXiv. 2025 Sep 11:arXiv:2509.09892v1. Preprint. [Version 1] (PMC12440066)
Supplement: Supplementary file 1 [file NIHPP2509.09892v1-supplement-1.pdf]

Supplementary Materials for

What do the fundamental constants of physics tell us about life?

Pankaj Mehta*, Jane Kondev†

* Email: pankajm@bu.edu

†Email: kondev@brandeis.edu

Contents

1 Materials and Methods **S3**

1.1 Rydberg energy and Bohr radius S3

1.2 Bond energy from bond length S4

1.3 Calculation of mass yield of a self-replicator S4

1.4 Berg kinematic viscosity S5

1.5 Maximum rate constant for chemical reactions S6

1.6 Minimum barrier height for bond formation S7

1.7 Minimum doubling time S9

1.8 Membrane voltage, current, and power S11

List of Tables

S1 **Self-replicators are constrained by physical laws governed by fundamental physical constants.** A list of the fundamental constants and their numerical values relevant for understanding chemistry-based self-replicating systems. Units are meters (m), seconds (s), Joules (J), electron volts (eV), Coloumbs (C), kilograms (kg). S16

S2 **The qualitative behavior of self-replicators can be expressed in terms of characteristic physical scales.** The fundamental constants in Table S1, can be combined in various ways to write characteristic physical scales that constrain the properties of self-replicators. $\hbar = h/2\pi$ is the reduced Planck constant and all numerical values are calculated at room temperature ($T = 298K$). S17

1 Materials and Methods

Here we give derivations of the results in the main text.

1.1 Rydberg energy and Bohr radius

Here we reproduce Weisskopf's argument for computing the Rydberg energy Ry and the Bohr radius a_0. Consider an electron in a hyrodgen atom. We would like to determine the typical distance of this electron and its typical energy E. The energy of the electron at a distance r from the nucles and momentum p is given by

$$E = -\frac{1}{4\pi\epsilon_0}\frac{e^2}{r} + \frac{p^2}{2m_e}, \tag{S1}$$

where ϵ_0 is the vacuum electric permittivity, e is the electron charge, and m_e is the mass of the electron. We can approximate the expected momentum in terms of the distance using de Broglie's relation, $p = \hbar/r$. Substituting this into the expression above yields the energy of the electron as a function of distance

$$E = -\frac{1}{4\pi\epsilon_0}\frac{e^2}{r} + \frac{\hbar^2}{2m_e r^2}. \tag{S2}$$

We can now determine the typical distance a_0 between electron and the nucleus as the distance that minimizes this energy (i.e. $\left.\frac{\partial E}{\partial r}\right|_{a_0} = 0$).

Namely, we have that

$$\left.\frac{\partial E}{\partial r}\right|_{a_0} = -\frac{\hbar^2}{m_e a_0^3} + \frac{1}{4\pi\epsilon_0}\frac{e^2}{a_0^2} = 0 \tag{S3}$$

Solving for a_0 gives

$$a_0 = \frac{4\pi\hbar^2\epsilon_0}{m_e e^2} = \frac{\hbar}{m_e\alpha c}, \tag{S4}$$

where we have introduced the fine structure constant $\alpha \approx 1/137$, the speed of light c, and used

$$\alpha\hbar c = \frac{e^2}{4\pi\epsilon_0}. \tag{S5}$$

This length scale a_0 is called the Bohr radius and has a numerical value of $a_0 \approx 0.53$Å $\approx 5.3\times10^{-11}$ m. The typical energy scale, the Rydberg energy Ry, is given by

$$Ry = -E(a_0) = \frac{m_e e^4}{2\hbar^2(4\pi\epsilon_0)^2} = \frac{e^2}{8\pi\epsilon_0 a_0} = \frac{1}{2}\alpha^2 m_e c^2 \tag{S6}$$

and has a numerical value of $Ry \approx 13.6$ eV $\approx 2.18\times10^{-18}$ J. These quantities are the fundamental length and energy scales of atomic physics that we make use of in the main text and below.

1.2 Bond energy from bond length

We would like to also estimate the typical energy scale associated with a chemical bond. To do so, we introduce a numerical factor – or as Weiskopf calls it a "fudge factor" – that relates bonds lengths l_{bond} to the Bohr radius

$$l_{bond} = f_{bond} a_0. \tag{S7}$$

For many commonly found bonds in organic molecules, $f_{bond} \approx 3$. The typical energy E_{bond} associated with an electron confined to a distance l_{bond} is just given by substituting l_{bond} into Eq. S2. By explicit calculation or by comparing the result with the third term in Eq. S6, we have

$$E_{bond} \approx \frac{Ry}{f_{bond}}. \tag{S8}$$

Numerically, this corresponds to a bond energy of about 4.5 eV or 430 kJ/mol. This should be compared to the measured energies of C-H bonds of 413 kJ/mol or C-C bond of 348 kJ/mol.

1.3 Calculation of mass yield of a self-replicator

We would like to calculate Y, the mass yield per unit of energy consumed by a self-replicator. We assume that the the primary source of energy consumption is the formation of chemical bonds in order to self replicate organic matter. In other words, energy consumption is dominated by anabolism rather than catabolism. If an organism of dry mass M requires the formation of N_{bond} new bonds, then the mass yield is just

$$Y = \frac{M}{N_{bond} E_{bond}}. \tag{S9}$$

It is reasonable to assume that the number of bonds that have to be formed during self-replication is proportional to number of atoms: $N_{bond} \approx bN$, where b is the proportionality constant which is order 1. It can be be smaller if the replicator is getting large number of building blocks directly from the environment (e.g. in rich media where all amino acids are supplied we expect $b \sim 1/10$). If we denote the average mass number of an atom in the organism by A_{bio}, then we also have the relation

$$N_{bond} \approx bN \approx \frac{bM}{A_{bio} m_p}, \tag{S10}$$

where m_p is the mass of a proton. Substituting this in the equation above, we find that the total mass cancels and that

$$Y \approx \frac{A_{bio} m_p}{b E_{bond}} = \left(\frac{A_{bio} f_{bond}}{b} \right) \frac{m_p}{Ry} = \left(\frac{A_{bio} f_{bond}}{b} \right) \frac{2 m_p}{m_e c^2 \alpha^2} \tag{S11}$$

where we have used Eqs. S6 and S8. Numerically, substituting we have that

$$Y \approx \frac{A_b f_{bond}}{b} 8 \times 10^{-7} g/J \tag{S12}$$

1.4 Berg kinematic viscosity

As pointed out by Ed Purcell, a wide variety of liquids seem to exhibit a similar minimum kinematic viscosity ν_{min} of around $10^{-7} m/\mathrm{s}^2$ (*23*). Recently, Tranchenko and Brazkin provided a simple dimensional analysis argument relating this number to fundamental physical constants (*24*). The basis of this argument is the observation that kinematic viscosity has its origin in quantum mechanical forces. A simple argument makes this clear. The kinematic viscosity has units of diffusion ($\nu_{min} \sim [length]^2/[time]$) and measures the "diffusion of momentum" in a fluid.

At high temperatures when the substance is a gas, the diffusion of momentum is controlled by thermal scattering between particles. In this regime, increasing the temperature also increases the viscosity since this increases the velocity and scattering length. In contrast, at low temperatures when substances are cooled, the viscosity increases when the temperature is decreased (the opposite of the gaseous phase). The microscopic origin of this is that particle become kinetically trapped and rearrangements of particles is dominated by rare rearrangements due to thermal activation, a process that is empirically well modeled by the Arrhenius Law. For this reason, the minimum of the kinematic viscosity of a liquid occurs when particles are dense enough for repulsive effects originating in quantum mechanics to be relevant but not so crowded that they get trapped kinetically.

In this regime, the diffusion of momentum is governed by atomic length scales and frequencies. There is only one natural length scale, the Bohr radius a_0 (Eq. S4). To calculate the kinematic viscosity, we also need to find a characteristic frequency ω_D governing particle interactions. This frequency should clearly depend on the mass of the fluid molecules and can be calculate by modeling particle interactions as springs. The only energy scale in the problem is the Rydberg energy (Eq. S6). Using the relation between energy and displacement for a harmonic spring $E = \frac{1}{2} K x^2$, we can

calculate a characteristic "spring constant" for the problem as $K \approx 2Ry/a_0^2$ and a characteristic angular frequency,

$$\omega_D = \sqrt{K/m} = \sqrt{\frac{2Ry}{ma_0^2}}. \tag{S13}$$

Then, the typical viscosity in this regime follows from dimensional analysis and can be written as

$$\nu_{min} \sim \frac{\omega_D}{2\pi} a_0^2 \sim \frac{1}{2\pi}\sqrt{\frac{2Rya_0^2}{m}} \sim \frac{1}{2\pi}\frac{\hbar}{\sqrt{mm_e}}, \tag{S14}$$

where in the last line we have used Eqs. S4 and S6. It will be helpful to express the molecular mass in terms of the proton mass as $m = A_f m_p$. With this substitution we have that

$$\nu_{min} = A_f^{-\frac{1}{2}} \nu_{Berg} \tag{S15}$$

where we have defined the "Berg kinematic viscosity"

$$\nu_{Berg} \sim \frac{\hbar}{2\pi\sqrt{m_p m_e}}. \tag{S16}$$

Numerically, this gives $\nu_{Berg} \approx 4 \times 10^{-7}$ m^2/s. This is very close to observed minimum viscosity for a wide variety of liquids (*24*). For example, the kinematic viscosity of water at 100C is about 3×10^{-7} m/s^2.

1.5 Maximum rate constant for chemical reactions

We are interested in rate at which chemical reactions happen. Following Berg and Purcell (*22*), we will assume that reactions are diffusion limited. In this case, the rate k_{on} at which a substrate at a concentration c encounters a molecule of size R is given by

$$k_{on} = 4\pi DcR, \tag{S17}$$

where D is the diffusion constant of the substrate. The diffusion constant can be expressed in terms of the temperature, viscosity η, and the Stokes radius of the substrate particle r using the Stokes-Einstei-Sutherland equation which holds at low-Reyonlds number:

$$D = \frac{k_B T}{6\pi\eta r} \tag{S18}$$

We can relate the viscosity to the kinematic viscosity by the density of the fluid ρ_f,

$$\eta = \rho_f \nu = A_f m_p c_f \nu, \tag{S19}$$

where in the second equality we have used the fact the density ρ_f can be written a the fluid concentration c_f times the mass of the fluid molecules $A_f m_p$, which we express in units of the proton mass. Substituting the last two equations into Eq. S17 gives

$$k_{on} = \frac{2k_B T c R}{3\eta r} = \frac{2}{3} A_f^{-1} \left(\frac{c}{c_f}\right)\left(\frac{R}{r}\right)\frac{k_B T}{m_p \nu} \tag{S20}$$

An upper bound on the *fastest* possible on rate, k_{on}^{max}, at a temperature T can be found by replacing ν by the *minimum* kinematic viscosity ν_{min} (S15). This gives an upper bound for the on rate of the form

$$\begin{aligned} k_{on}^{max} &= \frac{2}{3} A_f^{-1} \left(\frac{c}{c_f}\right)\left(\frac{R}{r}\right)\frac{k_B T}{m_p \nu_{min}} \\ &= \frac{2}{3} A_f^{-\frac{1}{2}} \left(\frac{c}{c_f}\right)\left(\frac{R}{r}\right)\frac{k_B T}{m_p \nu_{Berg}} \\ &= A_f^{-\frac{1}{2}} \left(\frac{c}{c_f}\right)\left(\frac{R}{r}\right)\left[\left(\frac{m_e}{m_p}\right)^{\frac{1}{2}} \frac{4\pi k_B T}{3\hbar}\right] \end{aligned} \tag{S21}$$

The term in the bracket sets a natural scale for chemical kinetics. For a temperature $T = 300\text{K}$, this takes a numerical value of

$$k_{on}^{max} = A_f^{-\frac{1}{2}} \left(\frac{c}{c_f}\right)\left(\frac{R}{r}\right) 4 \times 10^{12} \text{s}^{-1}. \tag{S22}$$

In other words, the characteristic time unit of chemistry is picoseconds.

For cells, R and r are both set by the molecular scale and hence $R/r \sim 1$. For water, $A_f = 18$, $c_f = 55M$. Finally, the highest concentrations found inside cells are in the 100mM range (for example, glutamate). Thus, for cells, we can further estimate

$$k_{on}^{max} \approx 1 \times 10^9 \text{ s}^{-1}, \tag{S23}$$

as an upper bound on the maximum collision frequency in cells.

1.6 Minimum barrier height for bond formation

In the transition-state theory of chemical kinetics, the rate of a chemical reaction takes the simple form

$$k = k_{on} e^{\frac{-\Delta E}{k_B T}}, \tag{S24}$$

where k_{on} is an attempt rate that governs how often molecules collide (see Eq. S21) and ΔE is an energetic activation barrier equal to the difference in energies between the initial state and the transition state. For this reason, a central role is played by ΔE.

We will be interested in calculating k^{max} – the fastest rate at which a chemical reaction can proceed. For this reason, we are interested in calculating the minimum activation energy of the transition state, ΔE^{min}. In order to model this, we assume that we can think of a bond as a spring and associate ΔE^{min} with the energy cost of stretched bond in the transition state.

Denote the rest length of the bond is l_{bond}. Then the corresponding spring constant can be calculated from the bond energy: $K_{bond} = 2E_{bond}/l_{bond}^2$. During a chemical reaction, we assume that in the transition state a bond stretches from its rest length l_{bond} to a length $l_{bond} + \Delta l = l_{bond}(1 + \delta)$, where we have defined the dimensionless quantity $\delta = \Delta l / l_{bond}$. The energy cost of this stretching is

$$\Delta E^{min} = \frac{1}{2} K_{bond} (\Delta l)^2 = E_{bond} \left(\frac{\Delta l}{l_{bond}} \right)^2 = E_{bond} \delta^2. \tag{S25}$$

Substituting Eq. S8 gives

$$\Delta E^{min} = \left(\frac{\delta^2}{f_{bond}} \right) Ry = \left(\frac{\delta^2}{f_{bond}} \right) \frac{\alpha m_e c^2}{2} \tag{S26}$$

where f_{bond} expresses the bond length in terms of the Bohr radius: $l_{bond} = f_{bond} a_0$. For the choice $f_{bond} = 3$ and $\delta = 0.3 - 0.5$ – a reasonable choice for a wide variety of reactions, we get numerical values of

$$\Delta E^{min} \approx 0.4 - 1.1\text{eV}. \tag{S27}$$

At room temperature $T = 300K$ ($k_B T \approx 0.026$eV), this corresponds to an Arrhenius factor

$$e^{\frac{\Delta E}{k_B T}} \approx e^{15} - e^{40} \approx 1 \times 10^6 - 1 \times 10^{17} \tag{S28}$$

Notice, that the square dependence of ΔE on δ^2 makes kinetic rates extremely sensitive to this exact number since the Arrhenius factor is exponential in ΔE. Since we are interested in the minimum vale of ΔE^{min} we will assume that δ must be greater that 0.3 and hence involve at least a thirty percent stretch in bond lengths. For peptide bonds in a ribosome, $\Delta E \approx 0.6 - 0.8$ eV (*29*).

1.7 Minimum doubling time

Before a self-replicator can divide, it must first double its size. This takes both time and energy. There are two possibilities for what sets the minimum doubling time: (i) self-replication is kinetically limited and (ii) self-replication is energy-limited. When self-replication is kinetically limited, the minimum doubling time is set by the time it takes to reproduce molecules (anabolism). In contrast, when the self-replicator is energy limited, the rate limiting step for self-replication is extracting energy from the environment (catabolism).

Kinetically limited self-replicators:

We now calculate the minimum doubling time for a self-replicator that is kinetically limited. The fastest self-replicator is an organism where each molecule replicates itself. If a molecule on average has N atoms, then using minimum doubling can be written in terms of the maximum rate of a chemical reaction k^{max} as

$$T_{min} = \frac{N}{k^{max}} = \frac{N}{k_0^{max}} e^{\frac{\Delta E}{k_B T}}, \tag{S29}$$

where we have used Eq. S21 for k^{max}.

Let us evaluate this assuming that protein synthesis is the limiting kinetic step. Assuming every protein translates itself, N will equal the typical number of peptide bonds in a protein which gives $N \sim 400$. Using Eqs S21, at room temperature we have

$$k_0^{max} \sim A_f^{-\frac{1}{2}} \left(\frac{c}{c_f}\right)\left(\frac{R}{r}\right) 4 \times 10^{12} \mathrm{s}^{-1}. \tag{S30}$$

For chemical kinetics, the target and substrate radii R and r are the smae order of magnitude. The typical concentration c of amino acids is 100-300mM. For water, $A_f \approx 18$ and $c_f \approx 55M$. With these substitutions we have

$$k_0^{max} \approx 1 \times 10^9 \, \mathrm{s}^{-1} \tag{S31}$$

The exponential factor can be estimated from Eq. S27. We see that ΔE equal ranges from 0.4 eV – 1.1 eV so that $e^{\frac{\Delta E}{k_B T}} \approx e^{15} - e^{40} \approx 1 \times 10^6 - 1 \times 10^{17}$ (see Eq. S27). Substituting these expressions into Eq S29 we have

$$T_{min} \approx 1 \, \mathrm{s} - 10^{11} \, \mathrm{s} \tag{S32}$$

The large range is due to the exponential dependence of T on the transition state energy ΔE.

Energetically limited self-replicators:

We now consider the case where self-replication is limited by catabolism– the ability to harvest energy from the environment. This is the case for extreme environments such as nitrite reducers deep in the ocean crust. In this case, growth is limited by the ability of to extract energy from the environment.

We will assume that the bacteria are absorbing spheres of typical size R and that the electron donors are found at concentration c, which for nitrite reducing bacteria is typically in the nM range. We assume that bacteria can extract an energy ΔE per molecule. This is set by the chemistry of redox reactions and is typically of order $0.1 - 1eV$, or in terms of the Rydberg energy between $Ry/100 - Ry/10$. The rate at which bacteria can extract energy is then limited by the diffusion limited flux incident on the cell (Eq. S21):

$$k_{on}^{max} = A_f^{-\frac{1}{2}} \left(\frac{c}{c_f}\right) \left(\frac{R}{r}\right) \left[\left(\frac{m_e}{m_p}\right)^{\frac{1}{2}} \frac{4\pi k_B T}{3\hbar} \right], \tag{S33}$$

where A_f and c_f are the atomic number and concentration of the fluid, R is the size of the absorber, in this case assumed to be the entire surface area of the cell, and r is the size of solute molecules. The total power consumption of the bacterial cell is just $P^{max} = k_{on}^{max} \Delta E$.

The total energy needed to reproduce is the $E_{tot} = N E_{bond}$, where N is the number of bonds. We assume, as in Eq. S10 that $N_{bond} \approx bN \approx \frac{bM}{A_{bio} m_p}$, where $b \approx 1 - 4$ is the number of bonds per atom, A_{bio} is the atomic number of biomass, and $M \approx 10^{-12}$ g is the mass of a cell. The total time that a self-replicator will take is just

$$T_{min} = \frac{E_{tot}}{P^{max}} = \left(\frac{bM}{A_{bio} m_p k_{on}^{max}}\right) \frac{E_{bond}}{\Delta E} \tag{S34}$$

Substituting Eq. S33, for a self-replicator with mass M we have

$$T_{min} = b A_{bio}^{-1} A_f^{\frac{1}{2}} \left(\frac{c}{c_f}\right)^{-1} \left(\frac{R}{r}\right)^{-1} M \left(\frac{3\hbar}{4\pi k_B T \sqrt{m_p m_e}}\right) \frac{E_{bond}}{\Delta E} \tag{S35}$$

$$= b A_{bio}^{-1} A_f^{\frac{1}{2}} \left(\frac{c}{c_f}\right)^{-1} \left(\frac{R}{r}\right)^{-1} M \left(\frac{3\nu_{berg}}{2 k_B T}\right) \frac{E_{bond}}{\Delta E} \tag{S36}$$

For deep sea chemolithoautotrophs such as Ammonia-Oxidizing Archaea, the limiting source of energy are H_2 molecules dissolved in water that occur at a concentration of about 1 nM. For these bacteria, the relevant fluid is water so that $A_f \approx 18$ and $c_f \approx 55M$, $R \approx 1\mu$ m (the size of the cell), $r \approx 1$ nM is the size of solute molecules, $\Delta E \approx 0.77eV$, $M \approx 10^{-12}g$, and $A_{bio} \approx 10$. Plugging in these numbers we have

$$T_{min} \approx 4 \times 10^6 \text{ s}, \tag{S37}$$

or approximately 42 days.

Lithoautotroph bacteria in the deep sea crust are thought to have doubling times in the range of years or $\approx 3 \times 10^7$ s. This is about four orders of magnitude above ammonia oxidizers. This can be understood by noting these bacteria reduce H_2 which thought to be found at sub-nanomolar range $c \approx 10^{-10} - 10^{-12}$ M. Substituting into Eq S36, one finds $\tau_{min} \approx 4 \times 10^7 - 4 \times 10^9$ s, consistent with experiments (*18*).

1.8 Membrane voltage, current, and power

We now consider a cell with a membrane and the minimal energetic cost of maintaining a voltage across the membrane. We assume that the concentration of charged ions inside the cell, c_i, differs substantially from the concentration of ions outside a cell c_o. For example, in *E. coli*, K^+ ions are typically at a concentration of 300 mM in the cytoplasm of the cell, while in the environment (LB medium) 10 mM is the norm. Then, from standard thermodynamic arguments we know that the free energy difference for ions inside and outside the membrane due this concentration difference is

$$\Delta G = k_B T \ln\left(\frac{c_o}{c_i}\right), \tag{S38}$$

where z the charge of the ion. As the ions cross the membrane down the concentration gradient an electrical potential difference builds up. At equilibrium, the free energy difference ΔG is counteracted by electrical potential energy. If we denote the membrane voltage by V_m, then energy balance gives us the Nerst equation

$$V_m = \frac{\Delta G}{ze} = \frac{k_B T}{ze} \ln\left(\frac{c_o}{c_i}\right). \tag{S39}$$

The prefactor $\frac{k_B T}{e}$ is often called the thermal voltage and is equal to approximately 26 mV at room temperature ($T = 298$ K). This sets the scale of the voltage across the membrane of all cells. For

the case of an *E. coli* cell, the equilibrium voltage for potassium ions is estimated, based on Eq S39 to be $V_m = 25.7 \times \ln(10/300)\text{mV} = -90\text{mV}$; the range of membrane potentials for *E. coli* under physiological conditions is -80 to -140mV.

We can also calculate the conductance of the membrane. To do so, we note that charged ions with charge z move across the membrane primarily through ion channels, which are transmembrane proteins that provide safe passage for ions by creating a water-like electrical environment within the channel formed by the protein. Whether an ion channel is open or closed is tightly regulated by the cell which provides the cell with the means of controlling the electrical properties of its cytoplasm and the membrane voltage.

Even in the absence of ion channels a lipid membrane eventually lets ions pass through. Thermally generated pores in the membrane provide passage that is not blocked by the large energy barrier imposed by the oily nature of the membrane interior. For determining the maintenance energy we primarily focus on ions passing through thermally generated pores as a mechanism for loss of membrane voltage. To get at the power dissipated by ions passing through a pore we first estimate the conductance of such a pore and then the average number of such pores in a membrane of a cell.

Inside a membrane pore, the membrane potential V_m gives rise to an electric field $E = V_m/d_m$, where d_m is the membrane thickness. This electric field exerts a force on the particles which is counteracted by frictional drag. We can write this force balance equation as

$$zeE = \gamma v_i, \tag{S40}$$

where γ is a viscous drag coefficient and v_i is the average ion velocity. At low Reynolds number, the drag coefficient γ can be related to the viscosity through the Stokes relation $\gamma = 6\pi\eta r$, where r is the Stokes radius of the diffusing ions. Using Eq. S39 relating the η to the kinematic viscosity ν and $E = V_m/d_m$, the velocity of the ions can be expressed as

$$v_i = \frac{z}{A_f c_f r d_m} \frac{eV_m}{6\pi m_p \nu}, \tag{S41}$$

where A_f and c_f are the atomic number and concentration of the fluid respectively.

If we denote the cross-sections surface area occupied by the pore by S_p, the ion concentration

by c, the maximum total current across the membrane I^{max} is given by

$$
\begin{aligned}
I^{max} &= zecS_p v_i^{max} \\
&= \frac{z^2}{A_f}\left(\frac{c}{c_f}\right)\left(\frac{S_p}{d_m r}\right)\frac{e^2 V_m}{6\pi m_p \nu_{min}} \\
&= \frac{z^2}{A_f^{\frac{1}{2}}}\left(\frac{c}{c_f}\right)\left(\frac{S_p}{d_m r}\right)\frac{e^2 V_m}{6\pi m_p \nu_{Berg}} \\
&= \frac{z}{A_f^{\frac{1}{2}}}\left(\frac{c}{c_f}\right)\left(\frac{S_p}{d_m r}\right)\left[\ln\left(\frac{c_o}{c_i}\right)\right]\frac{ek_BT}{3\hbar}\sqrt{\frac{m_e}{m_p}},
\end{aligned}
\tag{S42}
$$

where in going to the second line we have used Eqs S15 for the minimum viscosity and S39 for the membrane potential. The maximum conductance of membrane pore G_p^{max} also follows from the expression above by noting that

$$
I^{max} = G_p^{max} V_m, \tag{S43}
$$

which yields

$$
\begin{aligned}
G_p^{max} &= \frac{z^2}{A_f}\left(\frac{c}{c_f}\right)\left(\frac{S_p}{d_m r}\right)\frac{e^2}{6\pi m_p \nu_{min}} \\
&= \frac{z^2}{A_f}\left(\frac{c}{c_f}\right)\left(\frac{S_p}{d_m r}\right)\frac{e^2}{2\hbar}\sqrt{\frac{m_e}{m_p}}
\end{aligned}
\tag{S44}
$$

To estimate the maximum conductance of a membrane pore we take $c_f = 55$ Mol for water and $c \approx 300$ mM. The membrane thickness is typically $d_m = 4$nm while we take the pore diameter to be 6Å, i.e., the size of a hydrated K^+ ion. This gives $S_p = 30$Å^2 for the pore cross section ares. The radius of a hydrated ion r is $r \approx 3 \times 10^{-10}$ m, and $\ln\left(\frac{c_o}{c_i}\right) \approx 3$, while for water $A_f \approx 20$. Plugging in these numbers gives the maximum current and conductance of a membrane pore $G_p^{max} \approx 200 \times 10^{-12} S$, $I^{max} = 20 \times 10^{-12} A$ For comparison, the typical current through open ion channels are in the few pico-Ampere range while their conductances are in the $10 - 100$ pS range, both within an order of magnitude of our estimates. Note that had we used the kinematic viscosity of water, which at room temperature is $10^{-6} m^2/s$ or about an order of magnitude larger than the (universal) minimum viscosity, both estimates of the conductance and the current would be an order of magnitude smaller and therefore even closer to the measured values.

Using the estimates for the maximum current and membrane voltage we can calculate the

maximum power released by ions moving across a membrane pore:

$$P^{max} = V_m I^{max} = \frac{1}{A_f^{\frac{1}{2}}}\left(\frac{c}{c_f}\right)\left(\frac{S_p}{d_m r}\right)\left[\ln\left(\frac{c_o}{c_i}\right)\right]^2 \frac{(k_B T)^2}{3\hbar}\sqrt{\frac{m_e}{m_p}} \tag{S45}$$

$$= \frac{1}{3}A_f^{-\frac{1}{2}}\left(\frac{c}{c_f}\right)\left(\frac{S_p}{d_m r}\right)\left[\ln\left(\frac{c_o}{c_i}\right)\right]^2 \frac{k_B T}{\tau_{min}(T)} \tag{S46}$$

Equation yields the estimate $P^{max} \approx 4 \times 10^{-12} W$ for the power expended by an ion current through a single membrane pore. Again, this is about an order of magnitude larger than the estimate we get if we use the actual kinematic viscosity of water instead of ν_{min}. To obtain an estimate of the maximum power expended by the cell to maintain the balance of ions inside and outside the cell, we still need to estimate the expected number of pores in the cell membrane, as each pore will require the cell to expend P_{max} energy to counteract the free energy expended by the ion current. To estimate the average number of pores we make use of the idea that the molecular-scale energy of interaction between lipids in a membrane is of the order of 0.3eV, about an order of magnitude less than the scale of the covalent bond energy which itself was a few times less than the Rydberg energy.

This energy scale of non-covalent bonding between molecules, follows from a simple model of a molecule as a dipole whose dipole moment is $p_d = e l_{bond}$ and assuming that the interaction between two molecules is a dipole-dipole interaction. If the typical distance between the interacting dipoles is l_{int} then the estimate of the non-covalent bond energy is

$$E_{int} = \frac{1}{4\pi\epsilon_0}\frac{p_d^2}{l_{int}^3} = \left(\frac{1}{4\pi\epsilon_0}\frac{e^2}{2a_0}\right)\frac{2a_0 l_{bond}^2}{l_{int}^3}\ . \tag{S47}$$

Using our previous estimate for $l_{bond} = f_{bond} a_0$ and a new estimate for $l_{int} = f_{int} a_0$ where f_{int} is about a three times the molecular scale, or about ten times the atomic scale (i.e.,$f_{int} \approx 10$), we arrive at the estimate

$$E_{int} \approx 0.3eV \tag{S48}$$

which is to be compared to the range of non-covalent bond interactions for biomolecules that spans 0.03 to 0.3 eV, from the weakest London forces to the strongest hydrogen bonds (*30*). The same estimate is obtained by using the areal modulus of a lipid members $K_A = 0.3$ J/m^2. To create a pore of area $S_p = 30$ Å^2, the energy required is $1/2(0.3\,\text{J/m}^2 \times 30\,\text{Å}^2) = 0.3$ eV, the factor of $1/2$ accounting for the two layers of the lipids in the bilayer.

With this energy scale in hand we can estimate the probability that a pore opens up an lipid bilayer. We estimate that the energy needed to pull two lipids in a layer to a distance l_{int} is E_{int}. Therefore the probability of such a separation between two lipids happening spontaneously is $p_{int} \approx e^{-E_{int}/k_B T}$. For a pore to form spontaneously across the bilayer two lipids in both layers have to seperate to a distance l_{int} at the same time, leading to an estimate:

$$p_{pore} = p_{int}^2 \approx e^{-2E_{int}/k_B T} \approx e^{-24} = 4 \times 10^{-11} \tag{S49}$$

Since the number of lipids in an *E.coli* membrane is $S_{mem}/\pi l_{int}^2$, with $S_{mem} \approx 6\mu\text{m}^2$, the total number of pores at any given time is

$$n_{pore} = \frac{S_{mem}}{\pi l_{int}^2} p_{pore} = 8 \times 10^{-4} \tag{S50}$$

in other words the chance of a single pore opening in the membrane of an E.coli cell is about one in a thousand. This implies that the energy loss due to ions leaking through a spontaneously formed pore is

$$P_{dorm} \approx 8 \times 10^{-4} \times P_{max} = 3 \times 10^{-15}\text{W/cell}. \tag{S51}$$

This estimate goes down by an order of magnitude if we use the value for the kinematic viscosity of water, instead of ν_{min}. We can also compare it to the measured maintenance cost in recent lab experiments, which are of order 10^3 ATP/s or 10^{-16} W per cell (*20*). Similar values of the maintenance cost were estimated based on measurements of metabolic waste from a variety of bacterial cells observed in different natural environments (*28*).

Table S1: **Self-replicators are constrained by physical laws governed by fundamental physical constants.** A list of the fundamental constants and their numerical values relevant for understanding chemistry-based self-replicating systems. Units are meters (m), seconds (s), Joules (J), electron volts (eV), Coloumbs (C), kilograms (kg).

Constant	Symbol	Value	
		(SI units)	(Alternative units)
Speed of light	c	3.0×10^{8} m·s^{-1}	
Planck constant	h	6.6×10^{-34} J·s	4.1×10^{-15} eV·s
Elementary charge	e	1.6×10^{-19} C	
Fine structure constant	α	7.3×10^{-3}	$\approx \frac{1}{137}$
Mass of electron	m_e	9.1×10^{-31} kg	
Mass of proton	m_p	1.7×10^{-27} kg	
Boltzmann constant	k_B	1.4×10^{-23} J·K^{-1}	8.6×10^{-5} eV·K^{-1}

Table S2: **The qualitative behavior of self-replicators can be expressed in terms of characteristic physical scales.** The fundamental constants in Table S1, can be combined in various ways to write characteristic physical scales that constrain the properties of self-replicators. $\hbar = h/2\pi$ is the reduced Planck constant and all numerical values are calculated at room temperature ($T = 298K$).

Physical Scale	Symbol	Expression	Value (SI units)	Value (Alternative units)
Bohr radius	a_0	$\frac{\hbar}{m_e c\alpha}$	5×10^{-11} m	
Rydberg energy	Ry	$\frac{1}{2}m_e c^2\alpha^2$	2×10^{-18} J	13.6eV
Thermal energy		$k_B T$	4×10^{-21} J	25.7meV
Min. dynamic viscosity (Berg Viscosity)	ν_B	$\frac{1}{2\pi}\frac{\hbar}{\sqrt{m_e m_p}}$	4×10^{-7} m^2·s^{-1}	
Min. time scale for chemical kinetics	$\tau_{min}(T)$	$\frac{\hbar}{k_B T}\sqrt{\frac{m_p}{m_e}}$	1×10^{-12} s	
Chemical assembly constant	Y_c	$\frac{2m_p}{m_e c^2\alpha^2}$	8×10^{-7} g/J	
